# Prediction of biomarkers for brain metastasis in nonsmall cell lung cancer based on transcriptome sequencing

**DOI:** 10.1097/MD.0000000000043483

**Published:** 2025-07-18

**Authors:** Liangting Tan, Xuesong Xiang, Qiyi Qian, Qikun Zhang, Qiuran Xu, Wenhong Qiu, Xiaoliang Zheng

**Affiliations:** a Zhejiang Key Laboratory of Tumor Molecular Diagnosis and Individualized Medicine, School of Laboratory Medicine and Bioengineering, Hangzhou Medical College, Hangzhou, China; b Department of Immunology, Jianghan University, School of Medicine, Wuhan, China; c Zhejiang Key Laboratory of Tumor Molecular Diagnosis and Individualized Medicine, Zhejiang Provincial People’s Hospital, Affiliated People’s Hospital, Hangzhou Medical College, Hangzhou, China.

**Keywords:** biomarkers, brain metastasis, LASSO regression, nonsmall cell lung cancer, transcriptome sequencing

## Abstract

**Background:**

Lung cancer is one of the most prevalent malignancies worldwide, and the metastasis of nonsmall cell lung cancer often leads to rapid deterioration of patient conditions, with brain metastasis (BM) being the most detrimental. The mechanisms underlying lung cancer brain metastasis remain incompletely understood.

**Objective:**

This study aimed to elucidate the molecular mechanisms of lung cancer brain metastasis and identify potential biomarkers and therapeutic targets.

**Methods:**

The high invasiveness of H1975-BM51 cells was verified using Western blotting, cell invasion assays, and the establishment of an nonsmall cell lung cancer brain metastasis mouse model. Transcriptome sequencing of H1975 and H1975-BM51 cells was conducted, followed by Least Absolute Shrinkage and Selection Operator regression and single-gene Gene Set Enrichment Analysis to screen key genes. Quantitative real-time PCR and Western blotting were employed to detect the expression levels of the AGO3 gene in H1975-BM51 cells.

**Results:**

Transcriptomic analysis revealed that the AGO3 gene contributes to lung cancer brain metastasis by negatively regulating hormone metabolic processes. Compared with parental H1975 cells, both mRNA and protein expression levels of AGO3 were significantly upregulated in highly invasive H1975-BM51 cells.

**Conclusion:**

This study identifies AGO3 as a potential biomarker and therapeutic target for lung cancer brain metastasis.

## 1. Introduction

Lung cancer is one of the most common malignant tumors worldwide and remains the leading cause of cancer-related mortality, with its incidence and mortality rates increasing annually.^[1]^ Lung cancer is broadly classified into small cell lung cancer and nonsmall cell lung cancer (NSCLC), with NSCLC accounting for ≈85% of all lung cancer cases.^[2–4]^ Brain metastasis (BM) is a frequent complication of NSCLC, occurring in 10% to 20% of patients at initial diagnosis and in 25% to 40% during the course of the disease.^[5]^ Metastasis is a major determinant of poor prognosis, with the brain being the most common site of metastasis. It is estimated that 30% to 50% of NSCLC patients develop BM during disease progression.^[6]^ The prognosis for NSCLC-BM patients is extremely poor, with a median overall survival time ranging from 1.5 to 9.5 months, largely due to the limited efficacy of current treatment options.^[7,8]^

Currently, tyrosine kinase inhibitors (TKIs) such as crizotinib and entrectinib are FDA-approved drugs for treating lung cancer BM. However, these therapies face limitations, including poor BBB penetration and acquired drug resistance.^[9]^ Recent studies have highlighted the critical role of sphingolipid metabolism in tumor immune evasion and metastasis. For instance, Pan et al^[10]^ demonstrated that metabolic reprogramming of sphingolipids, particularly through the accumulation of glycosphingolipids, promotes immune suppression in hepatocellular carcinoma by masking inflammatory signals on cancer cell membranes. Similarly, identified sphingolipid-related long noncoding RNAs as key regulators of glioblastoma progression and immune microenvironment remodeling, suggesting their potential as prognostic biomarkers and therapeutic targets.^[11]^ These findings underscore the translational relevance of metabolic pathways in cancer prognosis and treatment resistance.

Despite recent advances, the molecular mechanisms underlying BM remain incompletely understood, particularly the factors that promote the survival of NSCLC cells in circulation and their colonization in the brain. Emerging multi-omics approaches have provided novel insights into cancer subtyping and prognostic modeling. For example, integrative analyses of transcriptomic and radiomic data in glioblastoma multiforme have enabled the identification of high-risk subgroups with distinct survival outcomes and therapeutic vulnerabilities. Similarly, studies in head and neck cancer and glioblastoma multiforme have leveraged CISD2-associated subtypes to refine prognostic stratification, offering methodological support for Least Absolute Shrinkage and Selection Operator (LASSO)-based feature selection in heterogeneous datasets.^[12,13]^ Therefore, identifying biomarkers for lung cancer BM is crucial for discovering novel therapeutic targets.

The LASSO is a regression technique used for variable selection and regularization, enhancing the predictive accuracy and interpretability of statistical models. In LASSO, data values are shrunk towards a central point, facilitating variable selection and parameter elimination. By adding a penalty equal to the absolute value of the coefficient magnitude, some coefficients can be reduced to zero and ultimately removed from the model, leading to a more streamlined and interpretable model. Single-gene Gene Set Enrichment Analysis (GSEA) is a method that evaluates the association between a gene and a biological process by calculating the similarity score between the expression pattern of a single gene and predefined gene sets.

In this study, we analyzed transcriptome data from H1975 cells and their highly brain-metastatic variant, H1975-BM51 cells, to explore potential biomarkers and associated pathways in lung cancer BM. This research aims to provide new therapeutic targets and insights for the treatment of lung cancer BM.

## 2. Materials and methods

### 2.1. Cell culture, morphological observation, and reagent sources

The human NSCLC cell line H1975 was purchased from the National Collection of Authenticated Cell Cultures. To extract brain metastatic cells from human NSCLC cell line H1975, parental H1975 cells (1×10^6^ cells/mL) were intracardially injected into the left ventricles of male BALB/c nude mice (100 μL). When mice exhibit behavioral changes on day 45 to 50, sacrificing the mice, brain tissue containing metastatic tumor cells was minced and cultured in RPMI-1640 with 10% Fetal Bovine Serum and antibiotics. Subsequently, tumor cells were reimplanted into the left ventricle of new nude mice. This in vivo selection process was repeated for 5 rounds to establish the highly brain metastatic subline H1975-BM51. The highly invasive H1975-BM51 cell line was derived from H1975 cells by inoculating them into mice and collecting circulating tumor cells over 5 rounds, followed by STR genotyping for authentication. Cells were cultured in T25 flasks using RPMI-1640 medium supplemented with 10% fetal bovine serum (Moregate Biotech, AU), penicillin (5 µg/mL), and streptomycin (5 µg/mL). Cultures were maintained at 37°C in a humidified atmosphere of 95% air and 5% CO₂. Phase-contrast images were captured at specified time points using an optical microscope (Olympus, JP) to obtain representative morphological images.

### 2.2. H1975 and H1975-BM51 sample data and data integration

Total RNA was extracted from H1975 and H1975-BM51 cells using TRIzol reagent (Accurate Biotechnology, CN). RNA concentration and purity were measured, and the Expression Console software was used for RMA analysis to generate a quality control report. The NSCLC cell line dataset GSE4342 was downloaded from the Gene Expression Omnibus database (https://www.ncbi.nlm.nih.gov/gds). Data from 2 H1975 cell line samples in the GSE4342 dataset were extracted using the R package Gene Expression Omnibus query (version 2.68.0). Transcriptome data from H1975 and H1975-BM51 cells were merged with the GSE4342 dataset using the R package inSilicoMerging. Batch effects were removed using the removeBatchEffect function from the R package limma (version 3.42.2), resulting in an expression matrix for lung cancer and lung cancer BM samples.

### 2.3. Western blot analysis

Total protein was extracted from both cell lines using lysis buffer, and protein concentration was quantified using the bicinchoninic acid assay (BCA). Proteins were mixed with loading buffer in appropriate proportions and denatured at 100°C for 5 minutes in a metal bath. Protein samples were separated by sodium dodecyl sulfate polyacrylamide gel electrophoresis (SDS-PAGE) and transferred onto a nitrocellulose membrane. The membrane was blocked with rapid blocking buffer for 20 minutes, followed by incubation with primary antibodies at 4°C overnight. The next day, the membrane was washed 3× with Tris-Buffered Saline with Tween (TBST) and incubated with secondary antibodies at room temperature for 1 hour. After 3 additional washes with TBST, the membrane was developed for signal detection.

### 2.4. Ethical statement

All animal experiments conducted in this study were reviewed and approved by the Animal Welfare and Ethics Committee of the Zhejiang Laboratory Animal Center (Approval No.: ZJCLA-IACUC-20010733).

### 2.5. In vivo imaging of mice

To monitor tumor BM, mice were intracardially injected with cells, followed by intraperitoneal injection of 100 µL of 15 mg/mL d-luciferin sodium salt solution. After injection, mice were anesthetized using 3% isoflurane. Once fully anesthetized, in vivo imaging was performed using an imaging system maintained at 1.5% isoflurane. The imaging parameters, including exposure settings, were adjusted, and images were acquired and saved in a predesignated folder.

### 2.6. Cell invasion assay

Cells were harvested from culture flasks and seeded at a density of 5 × 10⁴ cells per well into Transwell chambers precoated with Matrigel. After 48 hours of incubation, cells were fixed with methanol for 15 minutes and stained with crystal violet for 15 minutes. The chambers were allowed to dry, and images were captured.

### 2.7. Screening of DEGs

The R package limma (version 3.42.2) was used to identify differentially expressed genes (DEGs) between H1975 and H1975-BM51 samples. Genes with |logFC| ≥ 1.5 and *P* < .05 were considered significantly differentially expressed.

### 2.8. Prediction of biomarkers for lung cancer BM

LASSO regression was performed on the expression matrix of H1975 and H1975-BM51 samples using the R package glmnet (version 4.1-8). The optimal lambda value was determined through cross-validation. Model performance was evaluated using the area under the receiver operating characteristic (ROC) curve. Hub genes identified by LASSO regression were compared with DEGs using Venn diagrams to identify potential biomarkers. Univariate ROC analysis was conducted to assess the diagnostic potential of these biomarkers.

### 2.9. KEGG, GO, and single-gene GSEA analysis

Kyoto Encyclopedia of Genes and Genomes (KEGG) pathway annotations were obtained from the KEGG REST API (https://www.kegg.jp/kegg/rest/keggapi.html), and Gene Ontology (GO) annotations were retrieved using the R package org.Hs.e.g..db (version 3.1.0). Functional enrichment analysis was performed using the R package clusterProfiler (version 3.14.3). Single-gene GSEA analysis was conducted using GSEA software (version 4.3.3) with the c2.cp.kegg.v2023.1.Hs.symbols and c5.go.v2024.1.Hs.symbols datasets to explore the potential role of AGO3 in NSCLC and BM.

### 2.10. Quantitative real-time PCR

Cells were harvested when they reached 80% confluency. Total RNA was extracted using the RNAex reagent and an RNA extraction kit (Accurate Biotechnology). cDNA was synthesized using a cDNA synthesis kit (1st Strand cDNA Synthesis SuperMix for qPCR), yielding 20 µL of cDNA per reaction. Quantitative real-time PCR (RT-qPCR) was performed using glyceraldehyde-3-phosphate dehydrogenase as an internal reference. The relative expression of genes was calculated using the 2^−ΔΔCT^ method. Primer sequences were as follows:

AGO3 Forward: GGGCAAACCCATTAAACTGC
AGO3 Reverse: AACTGGTCTACGGTCTCCAA
GAPDH Forward: GGCTGAGAACGGGAAGCTTGTCAT
GAPDH Reverse: CAGCCTTCTCCATGGTGGTGAAGA

### 2.11. Statistical analysis

DEGs were identified using the R package limma (version 3.42.2) with thresholds of |logFC| ≥ 1.5 and *P* < .05. LASSO regression models were constructed using the glmnet package (version 4.1-8). Univariate ROC curves and area under the curve (AUC) values were generated using MetaboAnalyst 5.0 (https://www.metaboanalyst.ca/). Enrichment analysis with clusterProfiler was performed with a minimum gene set size of 5, a maximum gene set size of 5000, and thresholds of *P* < .05 and false discovery rate (FDR) < 0.25. Single-gene GSEA analysis was conducted using GSEA software (version 4.3.3). Differences between groups in PCR experiments were assessed using *t*-tests, with *P* < .05 considered statistically significant.

## 3. Results

### 3.1. Epithelial-mesenchymal transition, invasiveness, and NSCLC BM mouse model in high BM variants

**Figure 1.**
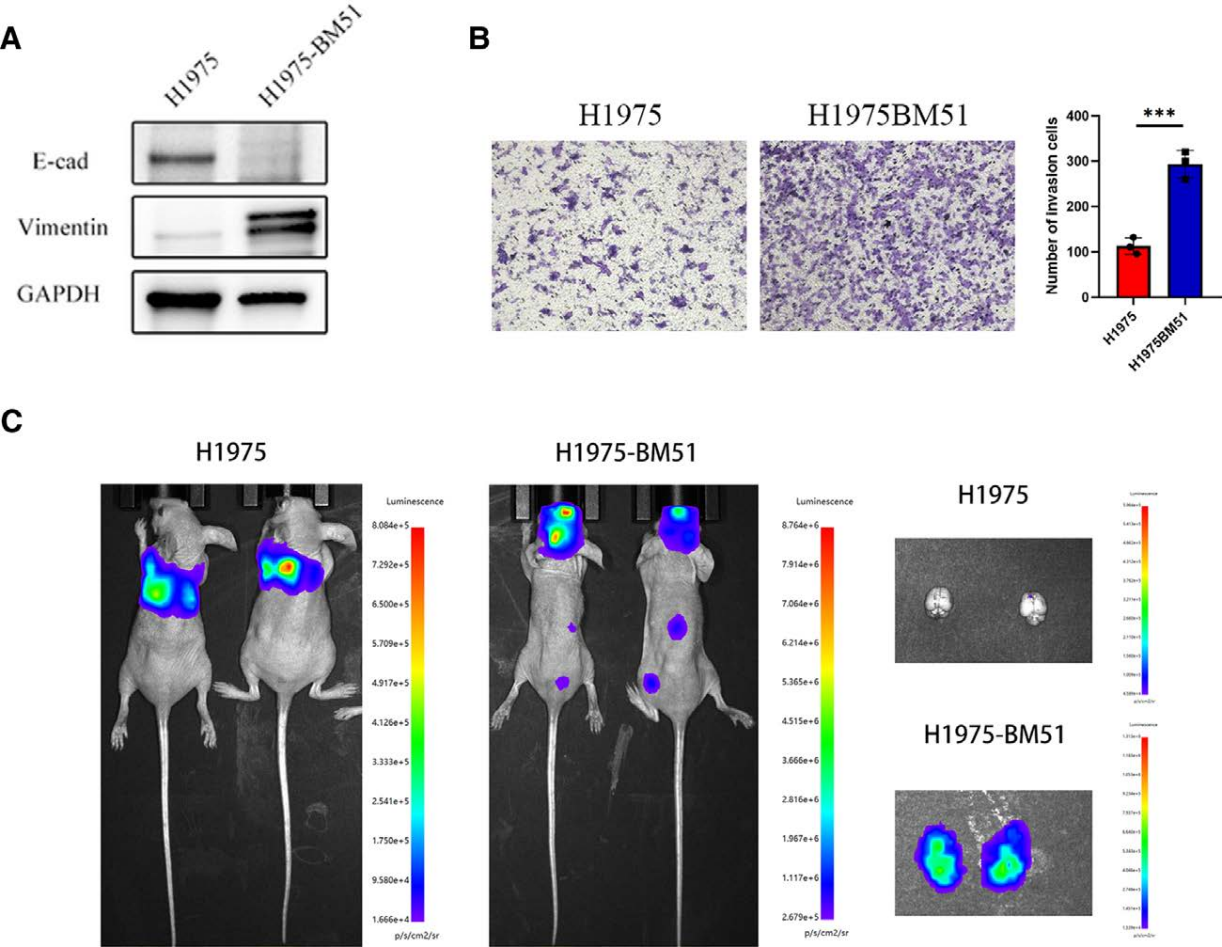
Characterization of brain metastatic potential in H1975-BM51 cells. (A) Expression of EMT-related proteins in H1975 cells versus H1975-BM51 cells. (B) Comparison of invasive capabilities between H1975 cells and H1975-BM51 cells. (C) In vivo imaging of mice inoculated with H1975 cells versus those inoculated with H1975-BM51 cells. EMT = epithelial-mesenchymal transition.

Western blot analysis was performed to validate the expression of E-Cadherin and Vimentin proteins. Compared to H1975 cells, H1975-BM51 cells exhibited significantly downregulated E-Cadherin expression and upregulated Vimentin expression, indicating that H1975-BM51 cells were in an epithelial-mesenchymal transition (EMT) state (Fig. 1A). Subsequently, the invasiveness of the 2 cell lines was assessed, revealing that H1975-BM51 cells demonstrated significantly higher invasiveness compared with H1975 cells (Fig. 1B). To evaluate BM capability, an NSCLC mouse model was established. Two weeks postinoculation, BM was observed in mice inoculated with H1975-BM51 cells, whereas no BM was detected in mice inoculated with H1975 cells (Fig. 1C).

### 3.2. Data integration and normalization of H1975 and H1975-BM51 datasets

**Figure 2.**
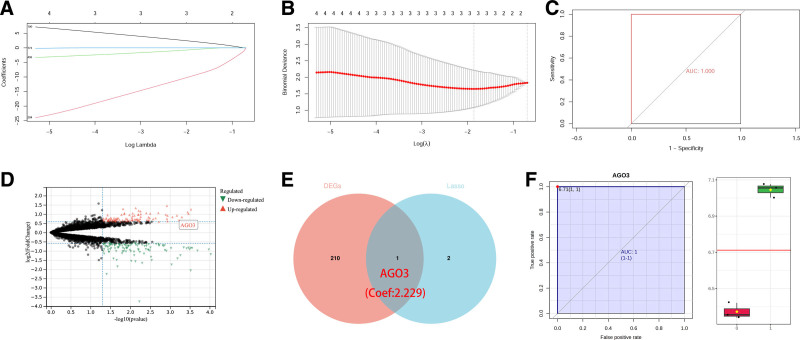
Transcriptomic analysis and model development for lung cancer brain metastasis biomarkers. (A) Density distributions of raw transcriptomic data (red), GSE4342-133a (blue), and GSE4342-133b (green) before and after normalization. (B) LASSO coefficient trajectories showing feature selection. The identified hub genes are AGO3, NCSTN, and PGK1. (C) Cross-validation curve for model optimization. (D) ROC curve of the LASSO regression model (AUC = 1, 95% CI: 1-1). (E) Volcano plot of differentially expressed genes (211 total: 99 up, 112 down; thresholds: |FC| > 1.5, *P* < .05). (F) Venn diagram overlap between DEGs (211) and hub genes (3), with AGO3 at intersection. (G) ROC curve for AGO3 alone (AUC = 1, 95% CI: 1-1). Inset shows expression distribution between cell lines. AUC = area under the curve, LASSO = Least Absolute Shrinkage and Selection Operator, ROC = receiver operating characteristic.

The 2 H1975 cell line datasets from GSE4342 were merged with the transcriptome data, and normalization was performed. The results are shown in Figure 2A. The transcriptome data are represented in red, GSE4342-133a in blue, and GSE4342-133b in green. After batch effect removal, the data distributions became consistent across all datasets.

### 3.3. LASSO regression and differentially expressed gene analysis

A LASSO regression model was constructed using the expression matrix of H1975 and H1975-BM51 cells, identifying AGO3, NCSTN, and PGK1 as hub genes associated with lung cancer BM (Fig. 2B and 2C). The LASSO regression model demonstrated excellent predictive performance (AUC = 1, 95% confidence interval: 1–1; Fig. 2D). Differential gene expression analysis of the H1975 and H1975-BM51 expression matrix revealed 211 DEGs, including 99 upregulated and 112 downregulated genes (Fig. 2E). Comparison of hub genes with DEGs showed that AGO3 expression was significantly upregulated in lung cancer BM and positively correlated with its occurrence (Fig. 2F). To further validate the potential of AGO3 as a diagnostic biomarker for lung cancer BM, univariate ROC analysis was performed, confirming its high diagnostic potential (AGO3 AUC: 1; Fig. 2G).

### 3.4. KEGG, GO, and single-gene GSEA analysis

**Figure 3.**
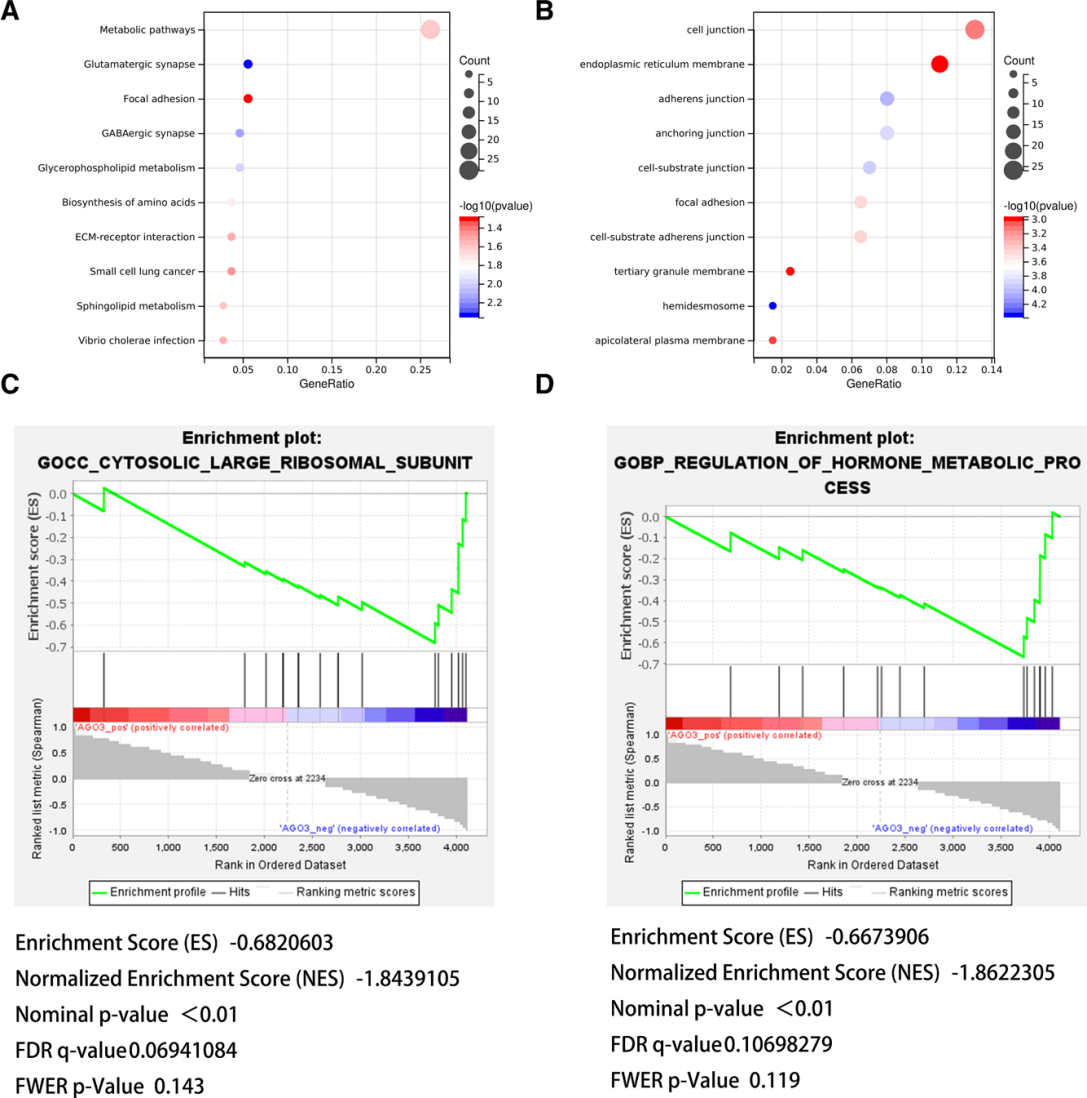
Functional enrichment analyses of differentially expressed genes and AGO3 characterization. (A) KEGG pathway analysis of DEGs between H1975 and H1975-BM51 cells (top 10 enriched pathways shown). (B) GO term enrichment of DEGs (biological process, molecular function, and cellular component categories). (C) Single-gene GSEA for AGO3 cellular component associations (Cytosolic large ribosomal subunit: NES = −1.84, FDR = 0.069, *P* < .01). (D) Single-gene GSEA for AGO3 biological process involvement (Regulation of hormone metabolic process: NES = −1.86, FDR = 0.107, *P* < .01). DEG = differentially expressed gene, FDR = false discovery rate, GO = Gene Ontology, GSEA = Gene Set Enrichment Analysis, NES = normalized enrichment score.

KEGG pathway analysis revealed that the DEGs between H1975 and H1975-BM51 cells were primarily associated with metabolic pathways. GO analysis indicated that these DEGs were predominantly involved in cell-extracellular matrix binding sites and endoplasmic reticulum membrane functions (Fig. 3A and 3B).

Single-gene GSEA analysis of AGO3 demonstrated that it was negatively correlated with the cytosolic large ribosomal subunit (enrichment score = −0.682, *P* < .001, FDR = 0.151) and the regulation of hormone metabolic processes (enrichment score = −0.667, *P* < .001, FDR = 0.111) (Fig. 3C and 3D). These findings suggest that AGO3 may play a role in the cytosolic large ribosomal subunit and participate in the negative regulation of hormone metabolic processes in lung cancer and lung cancer BM.

### 3.5. RT-qPCR and Western blot analysis of AGO3 expression

**Figure 4.**
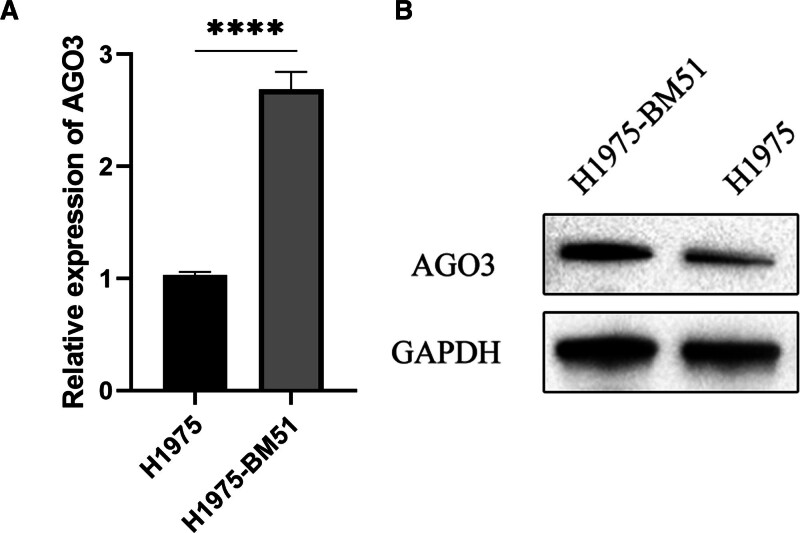
AGO3 expression in H1975-BM51 cells compared to H1975 cells. (A) Relative mRNA expression levels of AGO3 in parental H1975 cells versus highly invasive H1975-BM51 cells, as determined by RT-qPCR. (B) Protein expression levels of AGO3 in parental H1975 cells versus highly invasive H1975-BM51 cells. Data are presented as mean ± SD; *P < .05, **P < .01, ***P < .001. RT-qPCR = quantitative real-time PCR.

RT-qPCR results showed that AGO3 expression was significantly upregulated in H1975-BM51 cells compared with H1975 cells (Fig. 4A), consistent with the predictive analysis. Western blot analysis further confirmed that AGO3 protein levels were higher in H1975-BM51 cells than in H1975 cells (Fig. 4B), aligning with the predicted results.

## 4. Discussion

BM in lung cancer predominantly occur in the advanced stages of NSCLC and are associated with high incidence and poor prognosis.^[14–16]^ Approximately 10% of adenocarcinoma patients present with brain metastases at initial diagnosis,^[17]^ and in 50% of cases, the brain is the sole site of tumor recurrence (oligometastasis).^[18]^ Patients with BM face a dismal prognosis, with a 5-year survival rate of 2.9% and a high burden of neurological symptoms.^[19–21]^ Within the framework of the “seed and soil” hypothesis, the successful colonization of brain metastatic cells depends on specific properties that enable tumor cells to enter and survive in the brain.^[22,23]^ Recent advances in understanding the tumor microenvironment highlight its critical role in metastatic progression. A comprehensive review emphasizes how dynamic interactions between cancer cells, immune cells, and stromal components shape metastatic niches, particularly in immune-privileged sites like the brain.^[24]^ This framework supports our findings on AGO3, as its dysregulation may contribute to immune evasion and niche adaptation in BM.

The blood-brain barrier (BBB) and reduced chemosensitivity pose significant challenges, as most chemotherapeutic agents targeting primary tumors fail to inhibit BM.^[25–27]^ The BBB’s endothelial cells lack fenestrations and are reinforced by a basement membrane and astrocytic end-feet, creating a substantial barrier to the delivery of drugs to the central nervous system.^[28,29]^ ROS1 gene rearrangements lead to constitutively active fusion oncoproteins in ROS1-rearranged NSCLC, with studies showing that up to 36% of ROS1 fusion-positive NSCLC patients develop brain metastases at the time of advanced disease diagnosis.^[30,31]^

Current treatments for lung cancer BM include pharmacological therapies and surgery. TKIs such as crizotinib and entrectinib are FDA-approved drugs for treating lung cancer BM. Crizotinib, a small-molecule TKI targeting ALK, ROS1, and MET, has demonstrated significant antitumour activity in ROS1 fusion-positive NSCLC patients.^[32,33]^ Entrectinib, another TKI approved for ROS1 fusion-positive NSCLC, has shown tumor reduction in most patients, including those with brain metastases.^[34,35]^ However, these therapies are limited by poor BBB penetration and acquired drug resistance. Emerging evidence suggests that systemic immune-inflammatory responses influence metastatic progression and treatment resistance. For instance, pan-immune inflammation markers (e.g., neutrophil-to-lymphocyte ratio, platelet-to-lymphocyte ratio) have been linked to worse survival in NSCLC with BM.^[36,37]^ These findings align with our observation of AGO3 upregulation, as it may modulate immune-inflammatory crosstalk within the tumor microenvironment, further exacerbating metastatic aggressiveness.

Surgical interventions are costly, high-risk, and require patients to be in better physical condition. Immune checkpoint blockade and other methods remain under investigation, underscoring the importance of identifying biomarkers for lung cancer BM to develop novel therapeutic targets.

In this study, we validated the invasiveness and EMT status of the H1975-BM51 cell line, confirming its EMT state. Invasion assays revealed that H1975-BM51 cells exhibited significantly higher invasiveness compared with H1975 cells. A mouse model of NSCLC BM further demonstrated that intracardiac inoculation of H1975-BM51 cells led to BM, whereas H1975 cells did not, reinforcing the high metastatic potential of H1975-BM51 cells. Transcriptome sequencing data from H1975 and H1975-BM51 cells were analyzed using LASSO regression to construct a lung cancer-BM model, identifying AGO3, NCSTN, and PGK1 as hub genes. Differential gene expression analysis revealed 211 DEGs between the 2 cell lines, with 99 upregulated and 112 downregulated genes. Notably, AGO3 was significantly upregulated in H1975-BM51 cells.

To explore the mechanism of AGO3 in lung cancer BM, GO and KEGG analyses were performed on the DEGs, and single-gene GSEA was conducted for AGO3. GO and KEGG analyses indicated that the DEGs were primarily involved in cell-extracellular matrix binding sites, endoplasmic reticulum membrane functions, and metabolic pathways. Single-gene GSEA revealed that AGO3 functions in the cytosolic large ribosomal subunit and negatively regulates hormone metabolic processes, contributing to BM. RT-qPCR and Western blot experiments confirmed that AGO3 expression was significantly higher in H1975-BM51 cells compared with H1975 cells, supporting its potential as a biomarker.

AGO3, a member of the Argonaute protein family, plays a role in RNA interference and gene silencing. It binds to complementary microRNAs to suppress or degrade target mRNAs, and its dysregulation has been implicated in various cancers, including cervical, gastric, and prostate cancers, where it regulates cell proliferation and motility.

This study has some limitations, including a small sample size, though the results are statistically significant. Future work will expand the sample size and validate findings in clinical tissue samples. Additional experiments at the cellular and tissue levels are also planned.

In conclusion, this study identified AGO3 as significantly upregulated in H1975-BM51 cells compared with H1975 cells. AGO3 mediates lung cancer BM through the negative regulation of hormone metabolic processes, offering new insights and potential therapeutic targets for treating lung cancer BM.

## Acknowledgments

The authors gratefully acknowledge the contributions of the GEO database for providing valuable data resources essential to this study.

## Author contributions

**Conceptualization:** Liangting Tan.

**Data curation:** Liangting Tan.

**Investigation:** Liangting Tan.

**Methodology:** Liangting Tan.

**Software:** Liangting Tan.

**Validation:** Liangting Tan, Xuesong Xiang, Qiyi Qian, Qikun Zhang, Xiaoliang Zheng.

**Visualization:** Liangting Tan.

**Writing – original draft:** Liangting Tan, Xuesong Xiang.

**Writing – review & editing:** Liangting Tan, Qiuran Xu, Wenhong Qiu, Xiaoliang Zheng.

**Funding acquisition:** Xiaoliang Zheng.

**Supervision:** Xiaoliang Zheng.
